# Supplementary material for: Luminescence Properties of Epitaxial Cu2O Thin Films Electrodeposited on Metallic Substrates and Cu2O Single Crystals
Source: Materials (Basel). 2023 Jun 13;16(12):4349. doi: 10.3390/ma16124349 (PMC10301081; doi:10.3390/ma16124349)
Supplement: Supplementary file 1 [file materials-16-04349-s001.zip › materials-2385889-supplementary.pdf]

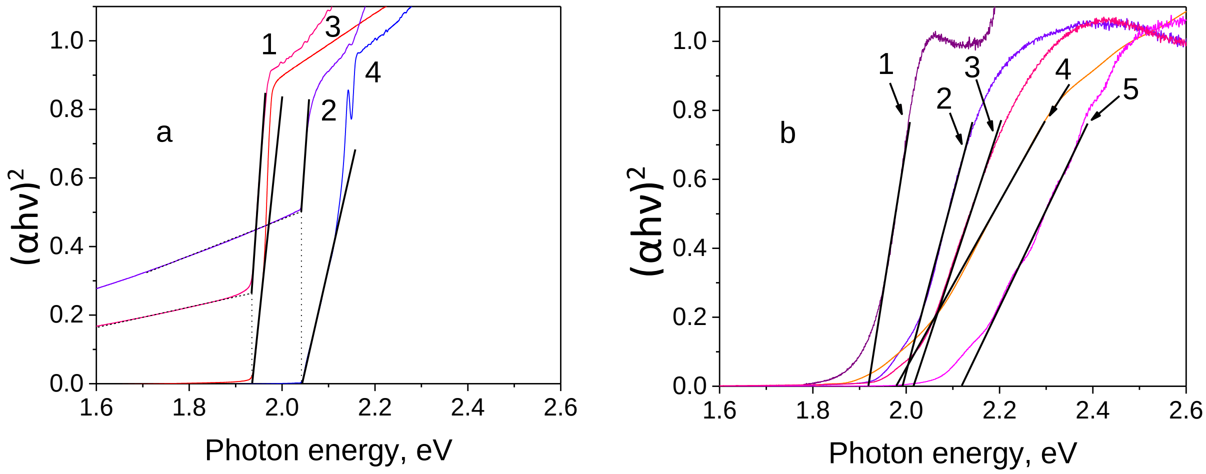

Figure S1. Tauc plot for (a) Cu_2O single crystals: 1 – SC (111) at RT, 2 – SC (111) at 10K, 3 – SC (100) at RT, 4 – SC (100) at 10 K; (b) Cu_2O thin films at RT: 1 – 111, 2 – 13, 3 – 40, 4 – 30, 5 – 39. SC (111) has an inclined ground level.

From the Tauc plot, the band gap value was determined as an intersection point of the tangent to a curve with an abscissa, or with an inclined ground level.

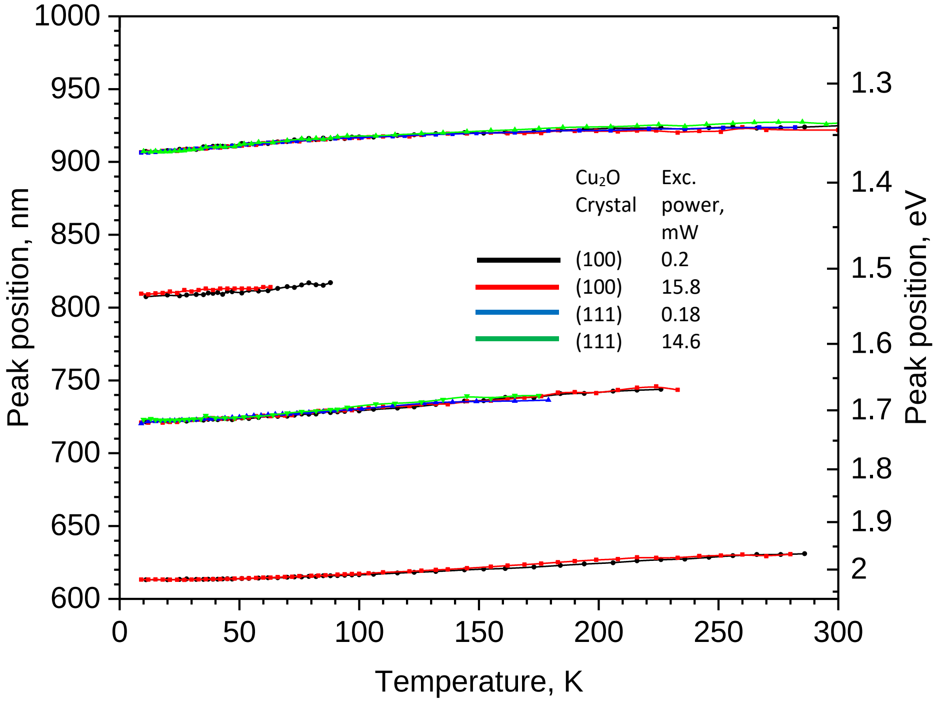

Figure S2. Peak position of the main emission bands of Cu_2O single crystals SC(100) and SC(111) under laser excitation power 0.2 and 15 mW in 10-300 K temperature range. Samples and excitation power are shown in the legend.

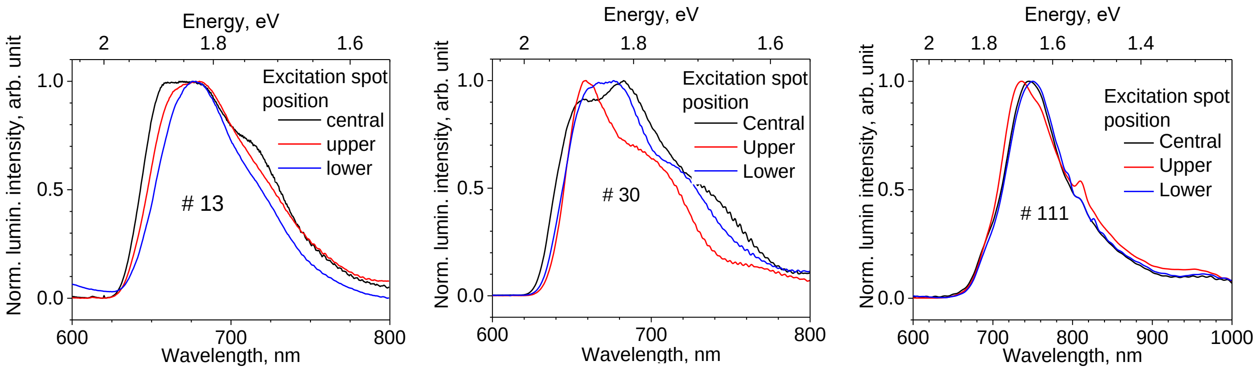

Figure S3. PL spectra of Cu_2O thin films 13, 30 and 111 at temperature 10 K, taken at different excitation spots.

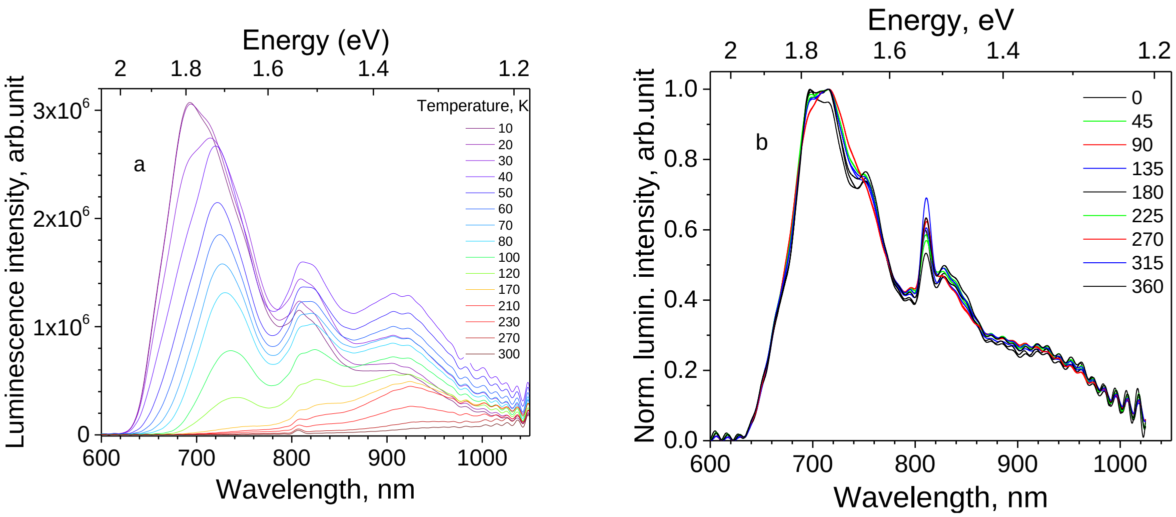

Figure S4. Spectral properties of Cu_2O thin film on Ag polycrystalline substrate (sample 39) under 532 nm laser excitation: (a) Thermal evolution of PL spectra (temperature values are shown on the graph); (b) polarized PL spectra at different angular positions of the analyzer at 10 K (angular degrees are shown on the graph).

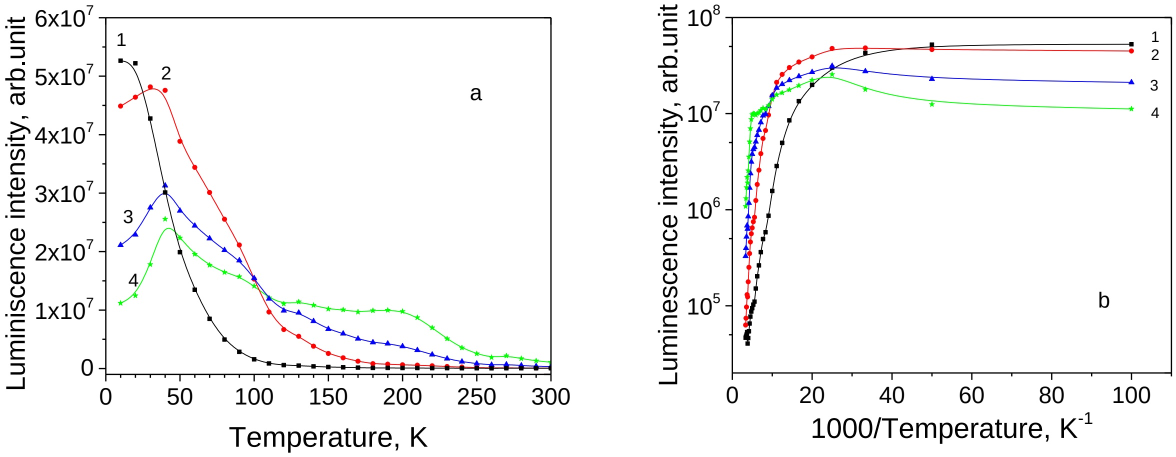

Figure S5. Thermal evolution of PL subbands for thin film sample 39: (a) PL intensity; (b) Arrhenius plot: 1 - 680 nm , 2 – 735 nm, 3 – 820 nm, 4 - 920 nm.

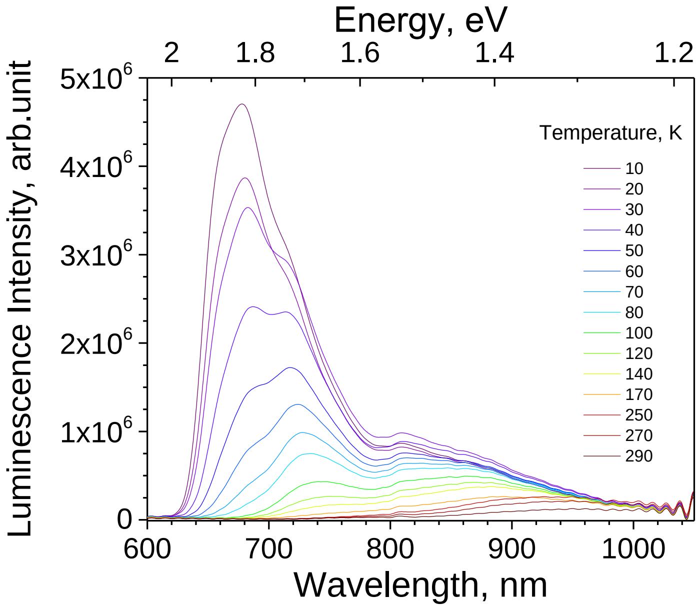

Figure S6. Thermal evolution of PL spectrum of Cu_2O thin film on Ag polycrystalline substrate (sample 40) under 532 nm laser excitation.

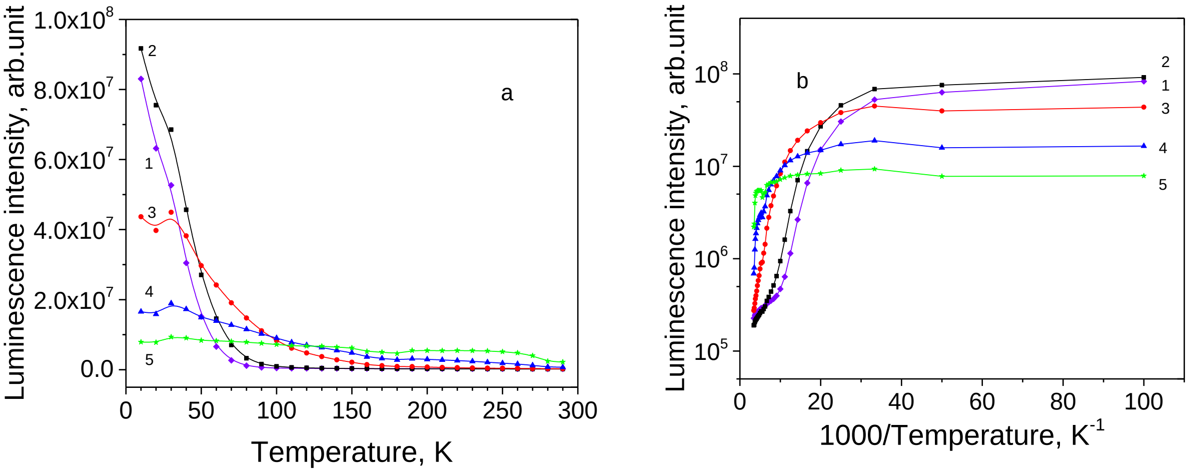

Figure S7. Thermal evolution of PL subbands for thin film sample 40: (a) PL intensity; (b) Arrhenius plot: 1 – 660 nm; 2 - 680 nm , 3 – 735 nm, 4– 820 nm, 5 - 920 nm.
